# The Evaluation of Eutectic Solvents as Catalysts for Mediating the Greener Synthesis of Poly(alkylene 2,5-furandicarboxylate)s

**DOI:** 10.3390/molecules31010077

**Published:** 2025-12-24

**Authors:** Beatriz Agostinho, Vinícius de Paula, Armando J. D. Silvestre, Andreia F. Sousa

**Affiliations:** CICECO—Aveiro Institute of Materials, Department of Chemistry, University of Aveiro, 3810-193 Aveiro, Portugal

**Keywords:** poly(alkylene 2,5-furandicarboxylate)s, eutectic solvents, polymer synthesis

## Abstract

In a quest to develop more sustainable polymers, decoupling their production from fuel-based resources and searching for alternative greener synthetic pathways are important priorities. Among the numerous polymers that fall into this category are furan-based polyesters. Besides the origin of polymers, their synthesis is another critical topic to consider regarding the overall greenness of the final product. However, despite several studies focusing on bio-based alternatives, such as poly(alkylene 2,5-furandicarboxylate)s, their synthesis still relies on air- and water-sensitive metal-based catalysts and is typically carried out under harsh conditions. This study explores an alternative approach with the application of eutectic solvents (ES) as catalysts for a more sustainable approach for synthesising furan-based polyesters, specifically poly(ethylene 2,5-furandicarboxylate) (PEF), as well as poly(trimethylene furandicarboxylate) (PTF) and poly(butylene furandicarboxylate) (PBF). Two different ES were evaluated as catalysts; the best results were obtained with urea and zinc acetate ES (U:Zn(OAc)_2_, 4:1 mol:mol). The resulting polymers were analysed for their structure, molecular weight, and thermal properties.

## 1. Introduction

Polymers have become an indispensable and valuable resource for our society, due to their unique properties, with global polymer production exceeding 410 Mt in 2023 [1]. The vast majority of these polymers (over 90%) are derived from fossil resources [1]. Therefore, redesigning polymers and decoupling them from non-renewable resources is an established priority in the pursuit of developing greener options, which has led to the current demand for bio-based polymers.

Among the numerous polymers that fit into this category, furan-based polyesters, such as those derived from 2,5-furandicarboxylic acid (FDCA), are among the most extensively studied [2], with poly(ethylene 2,5-furandicarboxylate) (PEF) being undoubtedly the most recognised [3]. This bio-based polymer is highlighted by its similarities to widely established fossil-based poly(ethylene terephthalate) (PET). Other FDCA-based polymers synthesised with longer-chain diols, such as poly(trimethylene 2,5-furandicarboxylate) (PTF) and poly(butylene 2,5-furandicarboxylate) (PBF), have also been attracting attention [4], due to their outstanding barrier properties, with PEF and PTF, for example, having a gas transmission rates of 0.03 and 0.17 cm^3^/m^2^·d·atm, respectively, in comparison with the 1.37 cm^3^/m^2^·d·atm rate observed for PET [5]. As a result, these polymers are being considered for future carbonated drink bottles [6,7].

Besides their origin, polymer synthesis is another critical topic to consider regarding the overall greenness of the final product. Indeed, the traditional synthesis of furan-based polyesters, typically through bulk polyesterification reactions, still relies on air- and water-sensitive metal-based catalysts, in addition to being carried out under harsh conditions, such as high temperatures (180–220 °C) [8,9]. Consequently, developing greener synthetic approaches is another requirement towards greener polymers. In the case of PEF, it is usually synthesised by a two-stage bulk polyesterification using 2,5-furandicarboxylic acid (FDCA) or its dimethyl ester derivative (DMFDC) and an excess of ethylene glycol at temperatures up to 220 °C and catalysed by metal catalysts such as antimony(III) oxide and titanium(IV) isopropoxide [9,10].

Alternative and greener approaches for synthesising PEF can include bulk polymerisation using organic non-metallic catalysts, like 1,8-diazabicylo[5.4.0]undec-7-ene (DBU) [11], which was used by Wu et al. to obtain PEF with an intrinsic viscosity of 0.54 dL·g^−1^, although at high temperatures of 170–240 °C and long reaction times of 11–13 h.

On the other hand, alternative and greener solvents, such as eutectic solvents (ES) [12], have been applied in different fields, including as catalysts for polymer synthesis [13,14]. ES are generally described as a mixture of two or more compounds that have a lower melting temperature than those of their individual components [15,16]. They are simple to prepare and do not require purification steps. ES have been reported to be used as alternative solvents, for example, in enzymatic polymerisation to enhance the enzyme’s activity and stability [17]. In furanic polyester synthesis, they can also be a greener alternative to organic solvents such as toluene or diphenyl ether [18]. Silvianti et al. [19] reported on two choline chloride-based ES as solvents for the enzymatic synthesis of furanic aliphatic polyesters with varying diol chain lengths (6 to 12). The best results were reported using choline chloride:urea (1:2 mol:mol) for the synthesis of poly(dodecamethylene-2,5-furanoate) (PDOF) with 41% yield and a molecular weight (M_w_) of 2700 g/mol.

In a different vein, and more recently, a urea:zinc acetate (U:Zn(OAc)_2_, 4:1 mol:mol) ES has been used as a catalyst for polyester depolymerisation, instead of only as a reaction solvent medium as previously mentioned [20,21,22], and particularly in our recent study on PEF chemical recycling [23]. This last study demonstrated the potential of ES to serve as catalysts in a closed-loop repolymerisation stage, leading to recycled PEF (rPEF) with a global yield of 69%.

Encouraged by the progress achieved, the present study aims to go beyond and explore, for the first time, the use of ES as catalysts for an eco-friendlier approach for the synthesis of FDCA-based polyesters and particularly of PEF, but also PTF and PBF. Two ES were assessed as catalysts, and the polymers’ structure, molecular weight, and thermal properties were investigated.

## 2. Results and Discussion

**Figure 1:**
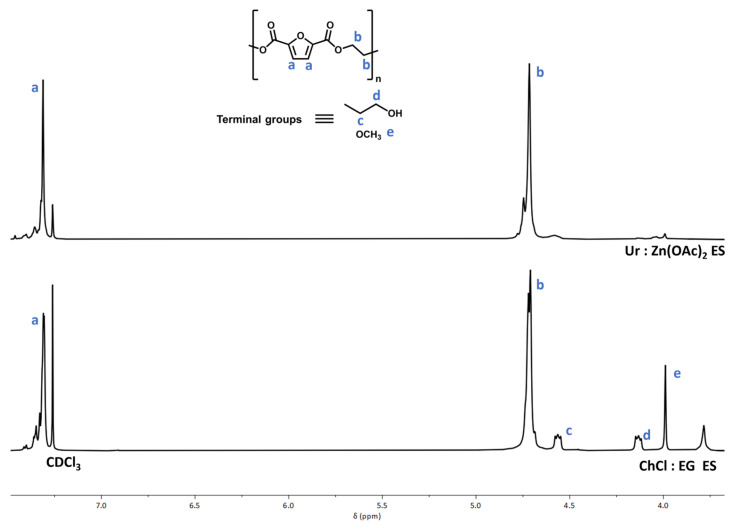
^1^H NMR spectra of PEF synthesised with different ES, U:Zn(OAc_2_) and ChCl:EG.

The first step of this study was dedicated to assessing the possibility of using a simple ES as the catalyst for the synthesis of PEF. Urea:zinc acetate (U:Zn(OAc)_2_, 4:1 mol:mol) was selected from previous evidence [23], and choline chloride:ethylene glycol (ChCl:EG, 1:2 mol:mol) was chosen due to the possibility of EG also acting as the monomer in polymer synthesis in the polyesterification reaction yielding PEF [24]. Reactions were carried out at 190 °C for 5 h, followed by 2 additional hours under vacuum at 220 °C. The obtained reaction isolation yield, the intrinsic viscosity of the isolated PEF ([η]), and the number-average degree of polymerisation (DP_n_) are summarised in Table 1. Based on ^1^H NMR analysis (Figure 1), it is possible to calculate the DP_n_ by considering the area of the proton resonances of the furan ring (a) and of the terminal group (d) [25].

From Table 1, it can be observed that both ES were able to mediate a polytransesterification reaction of PEF. The best ES in terms of PEF isolation yield, [η], and DP_n_ is U:Zn(OAc)_2_, with 77%, 0.2 dL·g^−1^, and 25, respectively. On the other hand, ChCl:EG did not perform as well, with only a 53% PEF isolation yield and low [η] and DP_n_ of 0.1 dL·g^−1^ and 10, respectively. Furthermore, control reactions were performed with each of the best ES components, namely urea and zinc acetate, with yields ranging from 16 to 66% and an average [η] of 0.15 dL·g^−1^.

For comparison, the results obtained with the standard metal-based catalyst used for PEF synthesis (titanium(IV) tert-butoxide, TBT) under the same reaction conditions show that the obtained yield is higher, around 87%, with an IV of 0.3 dL·g^−1^ and 75 DP_n_, which translates into a higher molecular weight overall. To evaluate the polymers’ chemical structure, the ATR FTIR, ^1^H, and ^13^C NMR spectra for PEF synthesised with the two different ES can be observed in Figure 1 and Figures S1–S3 in the Supplementary Information.

In Figure 1, ^1^H NMR resonances corresponding to the protons of the furan ring (a, 7.3 ppm) and to the methylene protons (b, 4.7 ppm) can be observed in both spectra, confirming PEF synthesis in both ES media. For PEF-synthesised ChCl:EG ES, two additional resonances corresponding to end-groups CH_2_CH_2_OH at 4.56 and 4.13 ppm (c and d, respectively), and an additional resonance around 3.99 ppm (e) attributed to the methoxy (OCH_3_) terminal group can be observed, which is in agreement with the obtained lower isolation yield and [η], which confirms the presence of an oligomer. Additionally, based on the ^1^H NMR analysis (Figure 1), it is also possible to calculate the number of repeating units of the depolymerisation product [25]: considering the area of the proton resonances of the furan ring (a) and of the OCH_3_ terminal group (e), it is possible to calculate a DP_n_ of 25 for PEF prepared in U:Zn(OAc)_2_ and only 10 for PEF oligomer prepared in ChCl:EG, which corroborates the obtained isolation yield and [η] values. The ^13^C NMR spectra (Figure S3, Supplementary Information) are in accordance with the expected chemical structure.

**Scheme 1:**
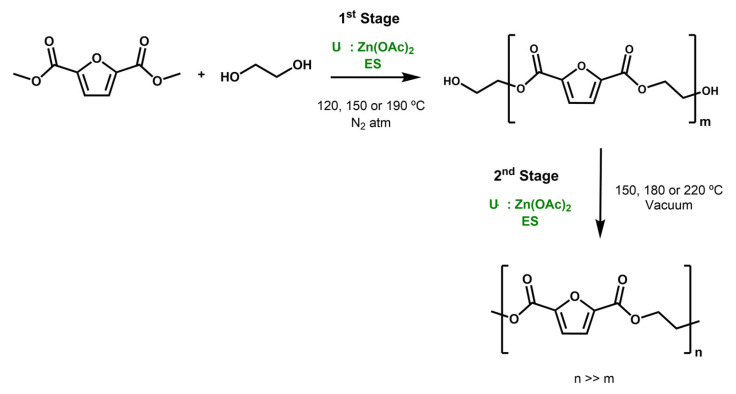
Reaction scheme of PEF synthesis using U:Zn(OAc)_2_ ES as a catalyst.

After the catalyst screening, U:Zn(OAc)_2_ media were selected for further optimisation of PEF synthesis conditions following Scheme 1.

Aiming to use milder synthesis conditions, lower reaction temperatures, ranging from 120 to 180 °C, were tested. The minimum temperature was set at 120 °C, as the DMFDC melting point is approximately 112 °C. Reaction temperature variation was investigated. In addition to the standard conditions (190–220 °C), two lower sets of temperatures (150–180 °C and 120–150 °C) were also tested. The applied conditions and the obtained results are summarised in Table 2.

**Figure 2:**
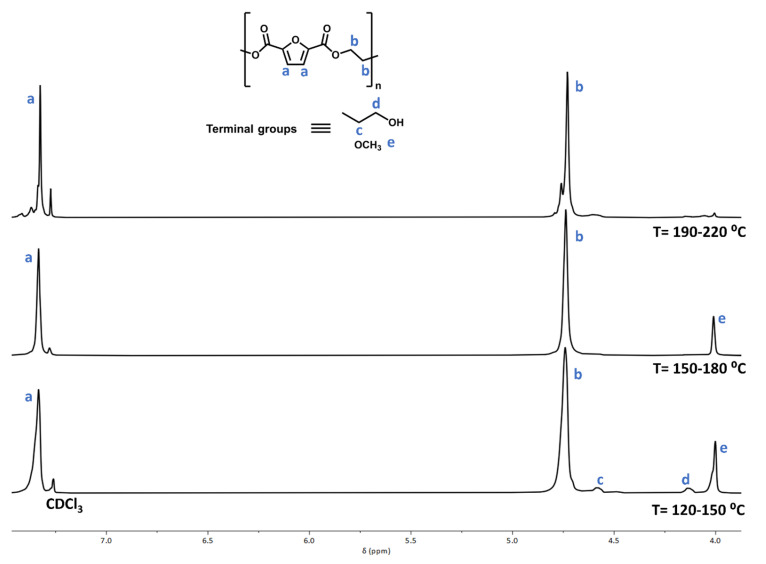
^1^H NMR spectra of PEF synthetized with U:Zn(OAc)_2_ ES (4:1) at different temperatures.

As expected, a trend can be observed whereby there is a lower yield and [η] for lower reaction temperatures. The lowest yield of 55%, an [η] of 0.1 dL·g^−1^, and a DP_n_ of only 9 were obtained for PEF synthesised at 120–150 °C. Under intermediate conditions of 150–180 °C, there is a slightly lower yield than under standard conditions (63% compared to 71%, respectively), an [η] of 0.2 dL·g^−1^, and a DP_n_ of 10. To evaluate their chemical structure, the ^1^H NMR of the obtained polymers can be seen in Figure 2.

In Figure 2, the ^1^H NMR resonances corresponding to the protons of the furan ring (a, 7.3 ppm) and to the methylene protons (b, 4.7 ppm) can be observed in the corresponding spectra. For lower temperatures (namely, 150–180 °C and 120–150 °C), an additional resonance around 3.99 ppm (e) attributed to the methoxy (OCH_3_) terminal group can be observed. For the lowest temperatures (120–150 °C), two additional resonances corresponding to PEF end-groups CH_2_CH_2_OH at 4.58 and 4.14 ppm, respectively, were also observed. This is in agreement with previous results, where lower temperatures correspond to the lowest yield, [η], and DP_n_. For that reason, the PEF synthesised with the lowest set of temperatures (120–150 °C) was not further investigated.

Additionally, thermal analyses (DSC and TGA) were conducted to assess the thermal properties of the resulting polymers, and the results obtained can be seen in Table 3 and in Figure S4 in the Supplementary Information.

In Table 3, for milder synthesis temperatures of 150–180 °C, the obtained PEF has a T_g_ of 73.4 °C, which is slightly lower than the T_g_ reported for PEF (75–80 °C) [26]. The same happens to the crystallisation temperature of 140 °C compared to 165 °C. The melting point of 211 °C is within the reported range for PEF (210–215 °C) synthesised with higher temperatures and metal-based catalysts [26]. Regarding the decomposition behaviour, the PEF synthesised with milder temperatures has both higher T_d,5%_ and T_d,max_ (327 and 383 °C compared to 310 and 373 °C for 180–220 °C). When the synthesis temperature rises to standard values (190–220 °C), the thermal properties generally improve, showing higher T_g_ and T_c_ (80.6 and 170 °C, respectively). For synthesis using a metal-based catalyst (TBT) at a temperature range of 190–220 °C, the T_g_ of 82 °C, crystallisation at 162 °C, and melting around 214 °C are all within the values obtained using U:Zn(OAc)_2_. On the other hand, regarding the decomposition behaviour, T_d,5%_ and T_d,max_ are higher at 355 °C and 403 °C, respectively.

After proving its potential for catalysing PEF synthesis with comparable properties to a metal-based catalyst (TBT), U:Zn(OAc)_2_ was also tested for the synthesis of other furanic polyesters, namely PTF and PBF, under the standard conditions originally used for PEF (190–220 °C, for a total of 7 h). The obtained isolation yield, intrinsic viscosity, and DP_n_ for all polymers can be seen in Table 4.

As can be seen in Table 4, U:Zn(OAc)_2_ was also able to mediate the synthesis of both PTF and PBF, yielding isolation yields above 74%. The final PTF’s intrinsic viscosity is 0.4 dL·g^−1^ and it has a DP_n_ of 75. For PBF, these values are slightly lower at 0.2 dL·g^−1^ and 10, indicating that an oligomer was obtained. This preliminary data shows that the U:Zn(OAc)_2_ can catalyse the synthesis of furanic-based polyesters, although there is still room for specific improvement, especially in the case of PBF. For the synthesis of PTF and PBF, the longer-chain diols need higher temperatures to support excess diol removal, which could be achieved by increasing the reaction’s temperature during the vacuum stage.

**Figure 3:**
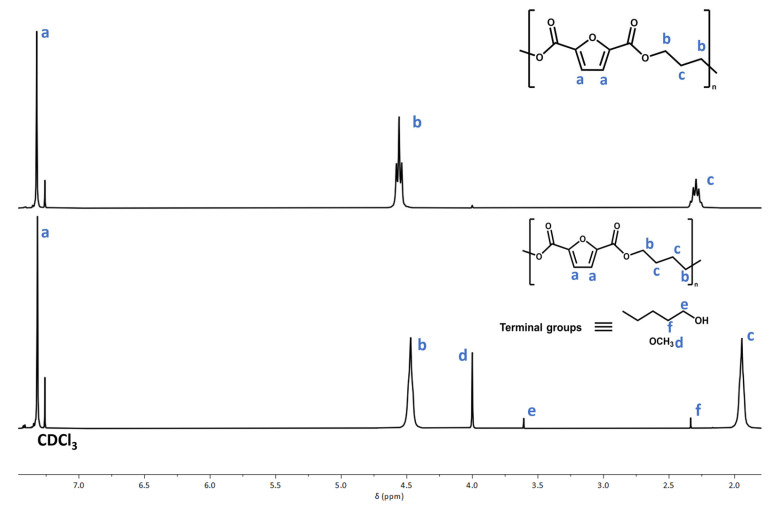
^1^H NMR of PTF and PBF synthesised with ES.

Furthermore, the structure of the resulting polymers was confirmed by ATR FTIR (Figure S5, Supplementary Information), ^1^H NMR (Figure 3 and Figure S6, Supplementary Information), and ^13^C NMR (Figure S7, Supplementary Information).

Observing Figure 3, in both spectra, the resonances corresponding to the protons of the furan ring (a, around 7.2 ppm) can be observed. For PTF, the methylene protons can be seen around 4.49 and 2.25 ppm (b and c, respectively), and for PBF, around 4.39 and 1.90 ppm (b and c). The terminal groups of PTF can also be seen around 3.78 and 2.01 ppm. These proton resonance assignments are in agreement with the reported ^1^H NMR data for PTF and PBF [5]. Similarly to PEF, the appearance of the DMFDC terminal group OCH_3_ around 3.99 ppm (d) can be observed for both polymers, which is consistent with the lower DP_n_ obtained for these polymers. Further characterisation is summarised in Table 5 and can be seen in Figure S8 in the Supplementary Information.

Analysing the obtained polymers’ thermal properties, for PTF, the glass transition temperature of 55 °C is in line with literature values (52 °C) [5]. The melting temperature is also within the reported values, with a slightly higher T_m_ of 174 °C compared to 169 °C [5,27]. In the case of PBF, the T_g_ of 26 °C is slightly lower than the one reported at around 37 °C. Similarly, the melting point is around 163 °C (170 °C in the literature), but the crystallisation temperature for PBF is similar, at 109 and 102 °C [5,27]. Regarding their degradation behaviour, for PTF, the maximum decomposition of 382 °C is within the reported range of around 386 °C, while for PBF, it is slightly lower at 389 °C compared to 407 °C [5].

## 3. Materials and Methods

### 3.1. Materials

Dimethyl furan 2,5-dicarboxylate (DMFDC, 99.9%) was purchased from Sarchem Laboratories (Farmingdale, NJ, USA). Urea (U, 99.0–100.5%), zinc acetate (Zn(OAc)_2_, 99.99%), choline chloride (ChCl, ≥99%), ethylene glycol (EG, 99.8%), 1,3-propanediol (PDO, 98%), 1,4-butanediol (BDO,), methanol (≥99.8%), chloroform (CHCl_3_, analytical grade), trifluoracetic acid (TFA, 99%), deuterated chloroform (CDCl_3_, 99.8 atom%D), deuterated trifluoracetic acid (CF_3_COOD, 99.5 atom%D), phenol (99%), 1,1,2,2-tetrachloroethane (TCE, ≥98%), and titanium(IV) tert-butoxide (TBT, 97%) were all purchased from Sigma Aldrich (St. Louis, MO, USA).

### 3.2. Eutectic Solvent Preparation

The different ES, urea:zinc acetate (U:Zn(OAc)_2_, 4:1 mol:mol) and choline chloride: ethylene glycol (ChCl:EG, 1:2), were prepared as described in the literature [21,23,24]. The ES components were briefly mixed according to the corresponding molar ratio at 70–100 °C for 2–8 h until they formed a clear, homogeneous liquid. The as-prepared ES were promptly used.

### 3.3. Polymer Synthesis Procedure

Poly(ethylene 2,5-furandicarboxylate) (PEF) was synthesised by a bulk polyesterification reaction adapted from elsewhere, using dimethyl 2,5-furandicarboxylate (DMFDC), an excess of ethylene glycol (molar ratio DMFDC:EG = 1:2.2), and the respective catalyst (ES) [27]. In the first stage, the temperature was gradually raised to 190 °C and kept at that temperature for 5 h. In the second stage, the reaction proceeded under vacuum, and the temperature increased to 220 °C in 2 h to remove excess ethylene glycol. Then, the reaction mixture was dissolved in chloroform (*ca.* 25 mL) with some drops of trifluoroacetic acid, and the polymer was precipitated by pouring the solution into an excess of cold methanol (*ca.* 500 mL), filtered, and dried. Control reactions were performed with each of the best ES components. Poly(trimethylene 2,5-furandicarboxylate) (PTF) and poly(butylene 2,5-furandicarboxylate) (PBF) synthesis was similarly carried out using 1,3-propanediol and 1,4-butanediol, respectively, with a molar ratio of DMFDC/diol = 1/1.2 [28].

### 3.4. Polymer Synthesis Optimisation

Polymer synthesis optimisation was performed for two additional sets of temperatures, 150–180 °C and 120–150 °C, for the reaction’s first and second stages, respectively. The remaining reaction conditions were kept constant as previously described (reaction time, catalyst, and DMFDC:EG molar ratio)

### 3.5. Polymer Characterisation

Attenuated total reflection Fourier transform infrared (ATR FTIR) spectra of the main products were obtained using a PARAGON 1000 Perkin Elmer FTIR spectrophotometer (Waltham, MA, USA) equipped with a single horizontal Golden Gate ATR cell. The resolution was 8 cm^−1^ at 64 scans, in the range 500–4000 cm^−1^. The ATR FTIR spectra are available in the Supplementary Information.

^1^H and ^13^C nuclear magnetic resonance spectroscopy (NMR) analyses of samples dissolved in CDCl_3_ with a few drops of CF_3_COOD were recorded using a Bruker AMX 300 Spectrometer (Billerica, MA, USA) operating at 300.13 MHz and 75.47 MHz, respectively. All chemical shifts were expressed as parts per million (ppm) downfield from tetramethylsilane used as the internal standard. Complete ^1^H and ^13^C NMR spectra are available in the Supplementary Information.

Intrinsic viscosity ([η]) measurements were carried out on an Ubbelohde-type viscometer maintained at 25 °C in a mixture of phenol/1,1,2,2-tetrachloroethane (50/50) (wt%/wt%). PEF was dissolved in that solvent mixture (0.1 g per 20 mL). The intrinsic viscosity was determined by the ratio of specific viscosity to PEF solution concentration (η_*sp*_/*c*, where η_*sp*_ = (*t*_1_ − *t*_0_) *t*_0_ and *t*_0_ and *t*_1_ are the solvent mixture elution time of the solvent mixture and polyester solution, respectively).

Differential Scanning Calorimetry (DSC) thermograms were obtained with a Netzsch Caliris 300 (Selb, Germany), and the thermograms were recorded following a heating rate of 2 °C/min (1st cycle), 20 °C/min (2nd cycle), and cooling rate of 20 °C/min under a nitrogen flow of 40 mL/min in a temperature range from 0 to 200 °C. Two heating/cooling cycles were repeated.

Thermogravimetric analyses (TGA) were carried out by a METTLER TOLEDO TGA 2 analyser (Columbus, OH, USA) equipped with a platinum cell using platinum pans to encapsulate the samples (*ca*. 5 mg). Thermograms were recorded under a nitrogen flow (50 mL/min) and heated at a constant rate of 20 °C/min from room temperature up to 800 °C.

## 4. Conclusions

This study demonstrates the potential of eutectic solvents (ES), especially urea and zinc acetate (U:Zn(OAc)_2_), as sustainable catalysts for synthesising furan-based polyesters such as PEF, PTF, and PBF. The results show that milder synthesis conditions with reaction temperatures from 150 to 180 °C can effectively produce PEF with desirable thermal properties. Additionally, PEF produced under milder conditions exhibited improved thermal stability, indicated by higher decomposition temperatures, although elevated synthesis temperatures (190–220 °C) further enhanced thermal characteristics, aligning with properties achieved using metal-based catalysts like TBT. The use of U:Zn(OAc)_2_ ES also proved efficient for synthesising furan-based polyesters with longer-chain diols, such as PTF and PBF, attaining yields above 74% and comparable intrinsic viscosities and molecular weights. These results highlight the possibility of eutectic solvents as environmentally friendly catalysts, enabling an alternative, efficient, lower-temperature production of furanic polyesters with promising thermal stability. Further studies expanding the range of both hydrogen bond donors and acceptors would be an important future step.

## Figures and Tables

**Table 1:** ES screening for PEF synthesis: polymer isolation yield and intrinsic viscosity.

ES	Isolation Yield (%)	[η]/dL·g^−1^	DP_n_^1^
U:Zn(OAc)_2_ (4:1 mol:mol)	71	0.2	25
ChCl:EG (1:2 mol:mol)	53	0.1	10

^1^ DP_n_ calculated through ^1^H NMR [25].

**Table 2:** PEF synthesis using U:Zn(OAc)_2_ (4:1) and different reaction temperatures.

Catalyst	Temperature/°C		Isolation Yield (%)	[η]/dL·g^−1^	DP_n_^1^
	1st Stage	2nd Stage			
U:Zn(OAc)_2_ ES	190	220	71	0.2	25
	150	180	63	0.2	10
	120	150	55	0.1	9

^1^ DP_n_ calculated through ^1^H NMR [25].

**Table 3:** Thermal data (obtained by DSC and TGA analyses) for PEF synthesised with U:Zn(OAc)_2_ ES at different reaction temperatures.

T_synthesis_/°C	T_g_/°C	T_c_/°C	T_m_/°C	T_d,5%_/°C	T_d,max_/°C
150–180	73.4	140	211	327	383
190–220	80.6	170	206	310	373

**Table 4:** PTF and PBF synthesis using U:Zn(OAc)_2_ ES as a catalyst, reaction yield, intrinsic viscosity, and DP_n_.

Polymer	Isolation Yield (%)	[η]/dL·g^−1^	DP_n_^1^
PTF	83	0.4	75
PBF	74	0.2	10

^1^ DP_n_ calculated through ^1^H NMR [25].

**Table 5:** Thermal data (DSC and TGA) of the different furanic polyesters synthesised with U:Zn(OAc)_2_ ES.

Polymer	T_g_/°C	T_c_/°C	T_m_/°C	T_d,5%_/°C	T_d,max_/°C
PTF	55	-	174	339	382
PBF	26	109	163	332	389

## Data Availability

The raw data supporting the conclusions of this article will be made available by the authors on request.

## Supplementary Materials

The following supporting information can be downloaded at: https://www.mdpi.com/article/10.3390/molecules31010077/s1. Figure S1: Complete ^1^H NMR spectra of PEF synthesised with different ES, U:Zn(OAc2) and ChCl:EG; Figure S2: FTIR spectra of PEF synthesised with different ES; Figure S3: ^13^C NMR spectra of PEF synthesised with different ES, U:Zn(OAc2) and ChCl:EG; Figure S4: TGA traces of PEF synthesised with U:Zn(OAc)_2_ ES at different reaction temperatures; Figure S5: FTIR spectra of PTF and PBF synthesised with ES.; Figure S6: Complete ^1^H NMR spectra of PTF and PBF synthesised with ES; Figure S7: ^13^C NMR of PTF and PBF synthesised with ES. Figure S8: TGA traces of PTF and PBF synthesized with ES.
