# Supplementary material for: The Evaluation of Eutectic Solvents as Catalysts for Mediating the Greener Synthesis of Poly(alkylene 2,5-furandicarboxylate)s
Source: Molecules. 2025 Dec 24;31(1):77. doi: 10.3390/molecules31010077 (PMC12786749; doi:10.3390/molecules31010077)
Supplement: Supplementary file 1 [file molecules-31-00077-s001.zip › molecules-4014388-supplementary.pdf]

molecules

MDPI

Article

The Evaluation of Eutectic Solvents as Catalysts for Mediating the Greener Synthesis of Poly(alkylene 2,5-furandicarboxylate)s

Beatriz Agostinho, Vinícius de Paula, Armando J. D. Silvestre, Andreia F. Sousa *

CICECO—Aveiro Institute of Materials, Department of Chemistry, University of Aveiro, 3810-193 Aveiro, Portugal.

* Correspondence: andreiafs@ua.pt

Supplementary Information

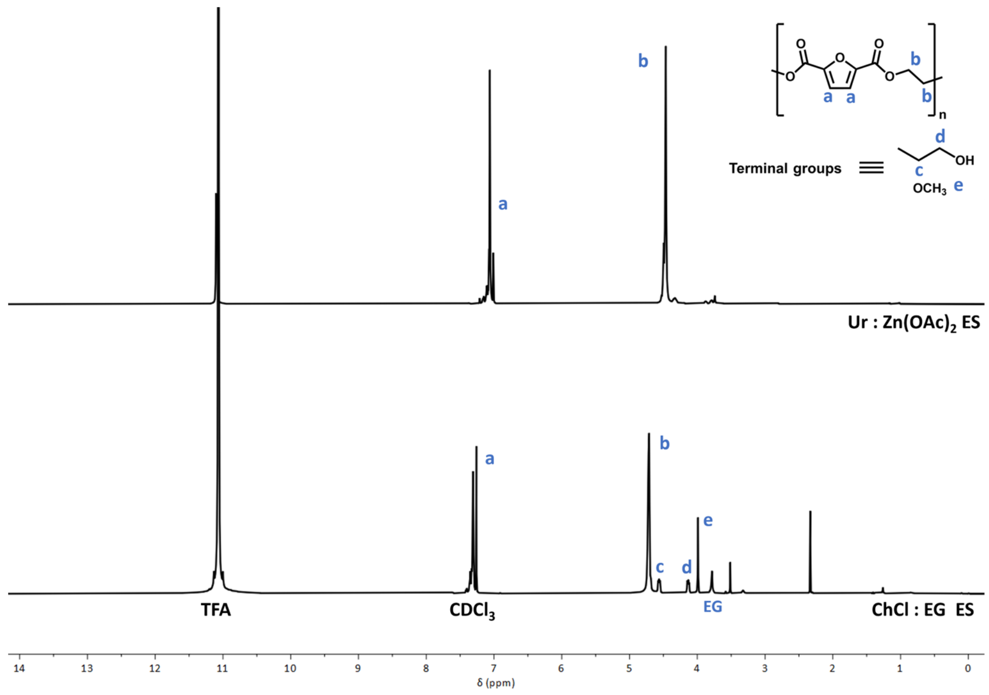

Figure S1 Complete ^{1}H NMR spectra of PEF synthesised with different ES, U : Zn(OAc2) and ChCl : EG.

 https://doi.org/10.3390/molecules31010077

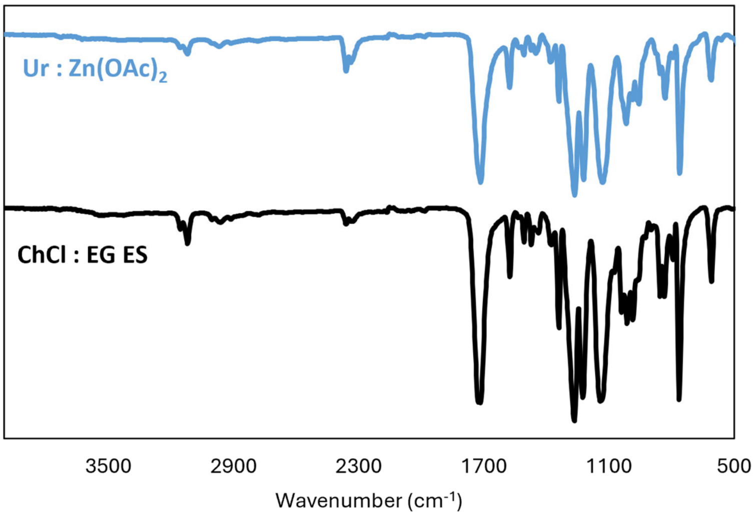

Figure S2 FTIR spectra of PEF synthesised with different ES.

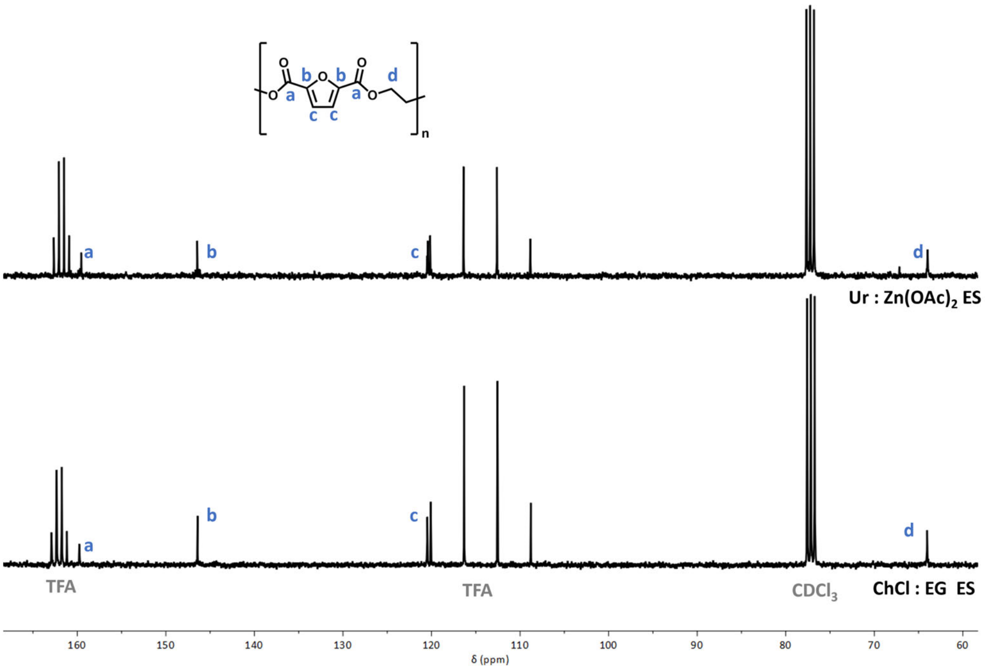

Figure S3 ^{13}C NMR spectra of PEF synthesised with different ES, U : Zn(OAc$_2$) and ChCl : EG.

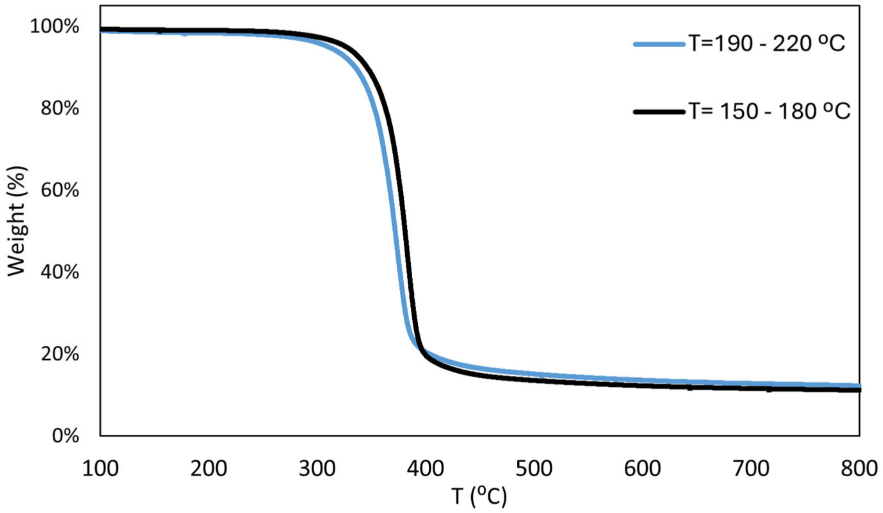

Figure S4 TGA traces of PEF synthesised with U : $Zn(OAc)_2$ ES at different reaction temperatures.

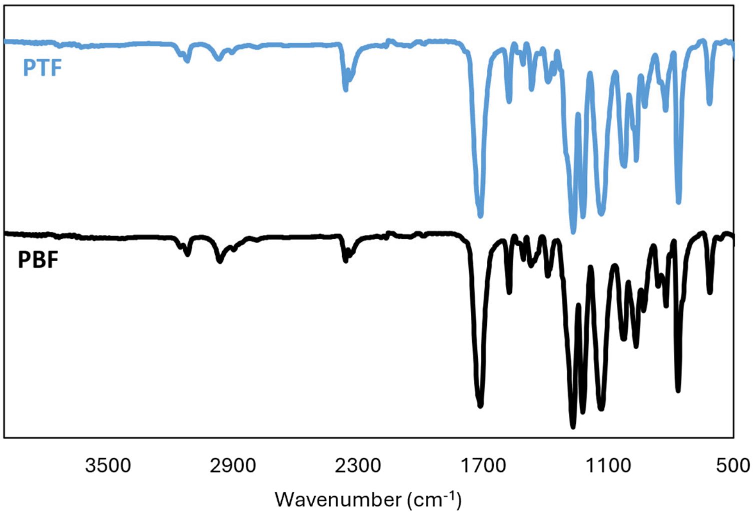

Figure S5 FTIR spectra of PTF and PBF synthesized with ES.

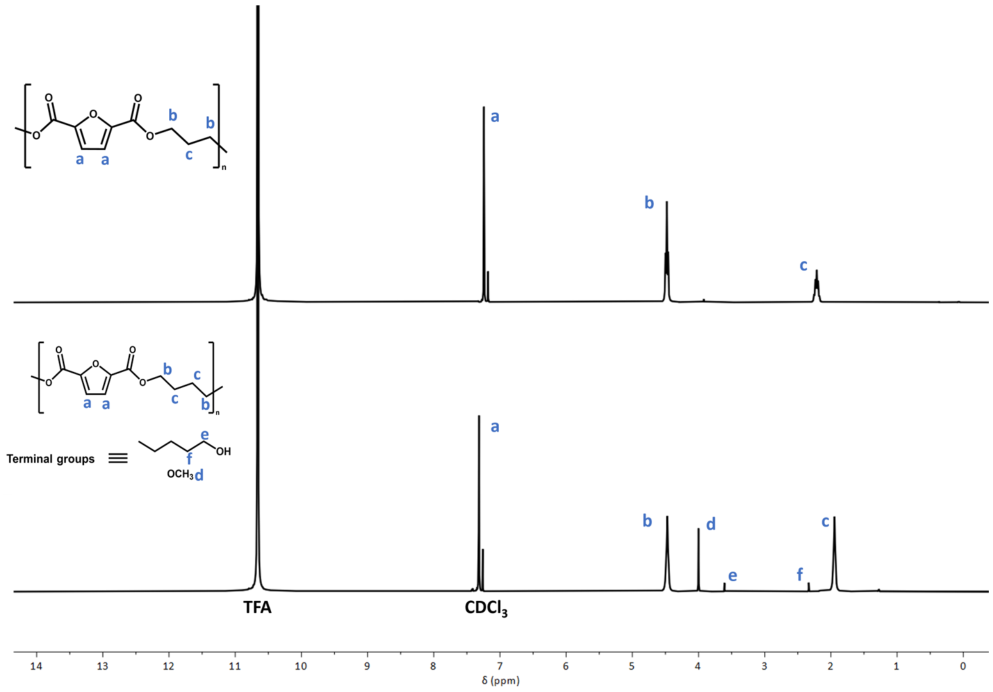

Figure S6 Complete ^{1}H NMR spectra of PTF and PBF synthesized with ES.

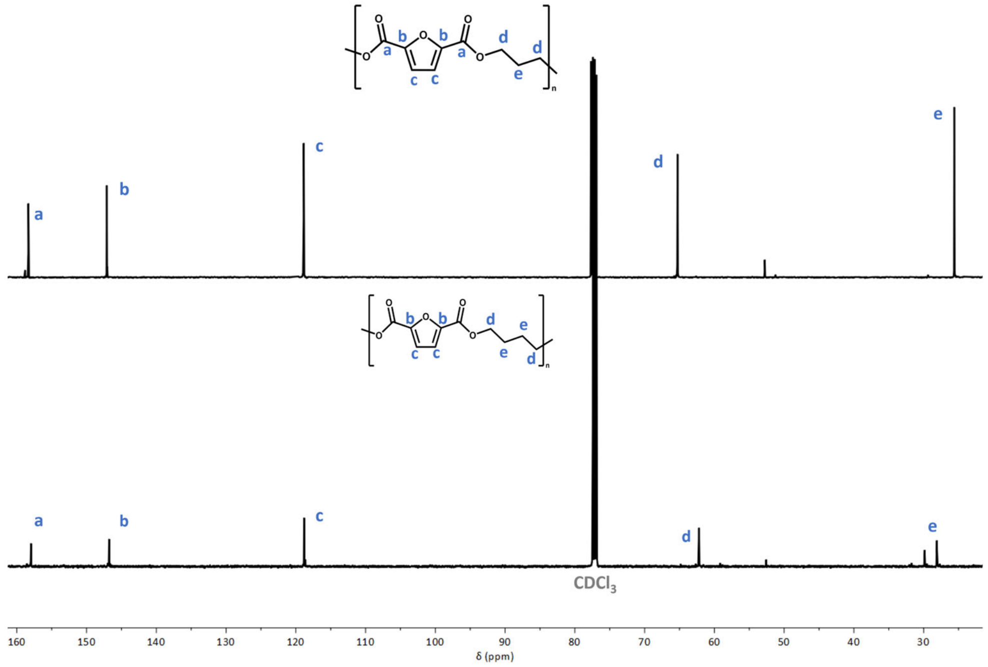

Figure S7 ^{13}C NMR of PTF and PBF synthesised with ES.

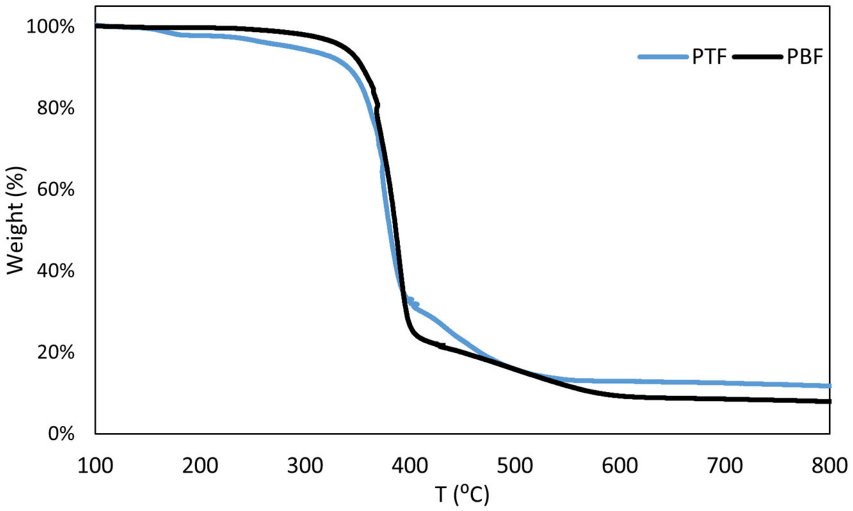

Figure S8 TGA traces of PTF and PBF synthesized with ES.
